# Determinants of the intention of elementary school nurses to adopt a redefined role in health promotion at school

**DOI:** 10.1186/1748-5908-5-93

**Published:** 2010-11-26

**Authors:** Guylaine Chabot, Gaston Godin, Marie-Pierre Gagnon

**Affiliations:** 1 Research Group on Behaviour and Health, Faculty of Nursing, Laval University, Québec, Canada; 2 Canada Research Chair on Behaviour and Health, Laval University, Québec, Canada; 3 Faculty of Nursing, Laval University, Québec, Canada

## Abstract

**Background:**

The quest for greater efficiency in the provision of primary healthcare services and the implementation of a "health-promoting school" approach encourage the optimal redefinition of the role of school nurses. School nurses are viewed as professionals who might be significant actors in the promotion of youth health. The aim of this study was to identify the determinants of the intention of elementary school nurses to adopt a new health-promotion role as a strategic option for the health-promoting school.

**Methods:**

This study was based on an extended version of the theory of planned behaviour (TPB). A total of 251 respondents (response rate of 70%) from 42 school health programs across the Province of Québec completed a mail survey regarding their intention to adopt the proposed health-promotion role. Multiple hierarchical linear regression analyses were performed to assess the relationship between key independent variables and intention. A discriminant analysis of the beliefs was performed to identify the main targets of action.

**Results:**

A total of 73% of respondents expressed a positive intention to accept to play the proposed role. The main predictors were perceived behavioural control (β = 0.36), moral norm (β *= *0.27), attitude (β = 0.24), and subjective norm (β *= *0.21) (*p*s < .0001), explaining 83% of the variance. The underlying beliefs distinguishing nurses who had a high intention from those who had a low intention referred to their feelings of being valued, their capacity to overcome the nursing shortage, the approval of the school nurses' community and parents of the students, their leadership skills, and their gaining of a better understanding of school needs.

**Conclusions:**

Results suggest that leadership is a skill that should be addressed to increase the ability of school nurses to assume the proposed role. Findings also indicate that public health administrators need to ensure adequate nurse staffing in the schools in order to increase the proportion of nurses willing to play such a role and avoid burnout among these human resources.

## Background

The quest for greater efficiency in the provision of healthcare services in industrialised countries encourages government authorities to review health professional roles [1]. International studies have shown that confusion regarding the role of school nurses and a lack of research regarding their effectiveness on the health and academic achievement of pupils have resulted in the need to question this role [2-12].

The studies also highlight the suboptimal use of school nurses in health promotion. In this respect, a recent study on nursing practices in health promotion concluded that public health nurses are far more active in the operational phase of health-promotion interventions and that they view themselves as support members of a team. Their supervisors, on the other hand, wish they would move towards what the authors refer to as a 'strategic role', becoming a person of influence with partnership skills able to work with a broad range of actors and increasingly involved in the planning and evaluation processes of health-promotion projects [13]. Financial cutbacks as well as a nursing shortage exert pressure on health-promotion roles, with the emphasis being placed on curative mandates, at the expense of health-promotion strategies. As a result, dissatisfaction among school nurses was noted regarding their roles [3,14]. Moreover, Brooks *et al.*[15] and Duplantie [3] indicated an urgent need to redefine the role of school nurses in light of new realities faced, such as increases in youth health problems. For example, between 1978 and 2004, combined rates of stoutness and obesity among young Canadians aged 12 to 17 years have more than doubled and the rate of obesity has tripled [16]. Multiethnicity, interprofessional, and intersectoral partnerships are other issues faced by school nurses [3,17]. It is also important to examine how school nurses can seize the opportunity of the health-promoting school (HPS) approach to redefine and expand their role to meet emerging demands [12,15].

Research on the redefinition and expansion of the role of school nurses in health promotion is scarce and often anecdotal. A review of the literature illustrates that in many industrialised countries, school nurses face similar realities with respect to youth health and their professional functions and conditions, whether employed by healthcare or school systems. Some researchers who have studied the role of school nurses suggest that these professionals should be included in decisions having an impact on their roles and responsibilities [3,18,19]. Therefore, this study addresses the viewpoint of school nurses because individual decisions are often central to the adoption of clinical-related behaviour and more information about the cognitive mechanisms underlying behaviour is needed to improve behavioural change interventions targeting healthcare professionals [20].

The purpose of this study was to identify the psychosocial determinants of the intention of elementary school nurses (ESNs) to adopt a redefined and expanded role in health promotion in the context of an HPS approach. In the present study, *adoption *refers to the acceptance of a role. This study addressed the following questions:

1. What proportion of ESNs intends to agree to play a redefined role in health promotion?

2. What psychosocial determinants from an extended version of Ajzen's theory account for an elementary school nurse's intention to agree to play a redefined role in health promotion?

3. What demographic factors (age, gender, education, number of years of practice as an ESN, employment status) account for an elementary school nurse's intention to agree to play a redefined role in health promotion?

### Theoretical background

Through a systematic review, Godin and colleagues [20] concluded that psychosocial theories are effective in understanding the cognitive mechanisms leading to the adoption of professional behaviour in healthcare. Eccles and colleagues [21] and Godin and colleagues [20] concluded that intention is a valid proxy measure for behaviour among clinicians, and the best prediction of intention was observed among samples of nurses. Nonetheless, few studies have focused on understanding the psychosocial determinants of healthcare professionals' intentions and behaviour. Among theory-based studies of healthcare professionals' behaviour, the TPB [22] and the theory of interpersonal behaviour (TIB) [23] have been used most often to date, and these theories have outperformed other psychosocial theories in the prediction of healthcare professionals' intentions and behaviour [20]. The TPB was preferred because it contains most of the variables recognised for their predictive capacity, such as control beliefs, social influences, normative beliefs, and consequences related to the adoption of a particular behaviour [20]. Moreover, TPB constructs are clearly defined and easy to operationalise and measure [24]. In addition, the TPB was formulated to take into consideration behaviour not always under volitional control [22,24]. ESNs are subject to the rules of their health unit.

According to Ajzen, individual behaviour is directly defined by an intention to adopt a particular behaviour. *Intention *refers to an individual's motivation regarding the performance of a given behaviour. The attitude towards the behaviour, the subjective norm, and perceived behavioural control are direct determinants of intention. As far as we know, this model has not been applied yet to the study of the redefinition of the role of school nurses. However, the TPB was successfully used to better understand the intention of public health nurses to adopt clinical behaviours [25-30]. Attitudinal factors and perceived behavioural control were the most important predictors of intention.

### Conceptual model

Following the recommendation of Perkins and colleagues [31], additional psychosocial constructs were added to the TPB model in order to gain a global understanding of the studied behaviour. With respect to factors explaining health professionals' intentions, Godin and colleagues [20] found that the most significant cognitive factors were beliefs about capabilities, beliefs about consequences, social influences, social/professional role and self-identity, and moral norm. In the literature, it is generally acknowledged that the TPB explains up to 40% of the variance in the prediction of intention [32]. The higher value reported in Godin and colleagues' [20] systematic review (59% explained variance) could possibly be related to the addition of other variables, such as Triandis's self-identity and moral norm. Self-identity helps to identify characteristics of the participants having the intention to adopt the proposed role. As suggested by Godin and colleagues [20], moral norm takes into consideration the ethical dimension of healthcare professionals' behaviour, and, as a single construct, it was found to be a frequent significant determinant of intention. This variable provides information on the moral obligation felt by the participants towards the proposed role. Finally, when a behaviour is performed in unstable or difficult contexts, conscious decision making is likely to be necessary to initiate and carry out the behaviour. Under these conditions, past behaviour (along with attitude and subjective norms) may contribute to intention [33].

## Method

### Studied population and sample

Health and social services centers (*Centres de santé et de services sociaux *[CSSSs]) that had a minimum of five ESN positions were recruited. This criterion is necessary to conduct a multilevel analysis that could be further realised. From the 50 CSSSs that met this criterion, 42 agreed to participate. The study population held part- and full-time ESN positions and included those on call for ESN position replacement. These on-call nurses had a minimum of six months of experience in the past year in school healthcare services in the province of Québec, Canada. School nurses work under the jurisdiction of local health and social services organisations, known as CSSSs. Data to estimate the number of positions per CSSS were obtained at the CSSS level, the only place where such data are recorded. Of the 358 mailed questionnaires, 256 were received. Among those received, three were returned uncompleted and two were completed by high school nurses. High school nurses have different mandates and different working conditions. Thus, their opinions would not reflect ESN realities. Consequently, 251 questionnaires were completed satisfactorily. Respondents were mailed Can$10 for compensation upon receipt of the completed questionnaire. The variation in response rate was generally homogenous across CSSSs, with a slightly higher proportion in smaller CSSSs. The average response rate of 70% served to meet the recommendations of Rashidian and colleagues [34] regarding sample size needed to predict intention based on the TPB.

### Data collection procedure

An authorisation to proceed with the survey was requested in a letter sent to the head office of every CSSS, also asking that a local contact person be identified. This person was usually the assistant to the school health program coordinator and was responsible for explaining the project to the ESN and distributing the consent form, questionnaire (see additional file 1), and the preaddressed and prestamped envelope to them. The prestamped envelope was to be mailed directly to the researcher with the completed questionnaire and signed consent form. One week later, each contact person distributed the recall letter sent by the researcher to all ESNs. This study was approved by all 21 ethical committees, including the local university, participating CSSSs, the Health and Social Ministry's Central Committee, and the Montréal Regional Public Health Unit.

### Instrument development and validation

#### Phase 1: Role definition

**Figure 1:**
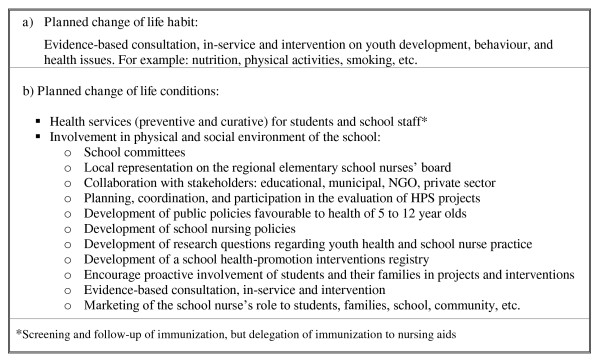
**A redefined health-promotion role for elementary school nurses**. ESN: elementary school nurse. HPS: health promotion. NGO: non-governmental organization. ESN: elementary school nurse. HPS: health promotion

The first step consisted of development of a vignette defining the role of the ESN (see Figure 1). The use of a vignette to study healthcare professionals' behaviour is recommended by Godin and colleagues [20] to better define the context of behavioural performance. For this task, we referred to the theoretical foundations of Hamric, Spross, and Hanson [35] as well as Sparacino [36] concerning the role of the specialist clinician, the role of the clinical nurse specialist in school health [37], the Schoenfeld [38] school nursing practicum, the Québec HPS approach, and the Beaudet *et al. *[13] strategic actor role. Moreover, this step was essential, considering the role discrepancy among ESN positions.

#### Phase 2: Telephone interviews

The questionnaire was developed following the guidelines of Ajzen [39]. The development of the instrument involves qualitative and quantitative approaches. The qualitative part consisted of obtaining information relevant to the study behaviour (*i.e.*, adopting the new nursing role) according to the emic-etic anthropological approach [40] recommended by Davidson, Jaccard, Triandis, Morales, and Diazguerrero [41]. The emic (*i.e.*, subjectivist/qualitative/insider) perspective was completed as an initial step by individual semistructured telephone interviews conducted among a convenience sample of 27 ESNs. An open-ended questionnaire comprising seven questions was used. The questions dealt with nurses' perceived pros and cons of the redefined role, barriers and facilitating conditions affecting their intention to adopt the proposed role, and individuals or groups favourable or unfavourable to their adoption of the proposed role.

#### Phase 3: Content analysis

The etic (*i.e.*, objectivist/quantitative/outsider) terms were performed following a content analysis to extract salient modal beliefs common to this population. The content analysis was performed independently by two researchers who agreed on the classification and labelling of themes extracted. The responses provided by more than 25% of the nurses were kept to form the items used to measure the belief-based variables of the TPB. Thus, the number of items forming each construct varied according to the number of popular responses given by the ESNs. The items measuring the main variables of the TPB (*i.e.*, attitude, subjective norm, perceived behavioural control) were developed according to [24] guidelines. Finally, a face-validity check was performed by two school nurse specialists involved in the HPS implementation approach.

#### Phase 4: Item development

Intention was measured by means of four items: '...if I had the choice, I would accept to play the proposed role' (seven-point scale: 7 = strongly improbable; 1 = strongly probable). Attitude was measured with four items composed of two pairs of adjectives, appearing after the sentence: 'To accept to play the proposed role would be...' (seven-point scale: 7 = very useful; 1 = very unuseful). Three items served to measure subjective norm: 'Most people who are important for me would recommend to accept to play the proposed role...' (seven-point scale: 7 = strongly agree; 1 = strongly disagree). Perceived control was measured by three items as follows: 'I would be able to play the proposed role' (seven-point scale: 7 = strongly improbable; 1 = strongly probable). Three items were used to measure moral norm: '...to accept to play the proposed role corresponds to my values'. Behavioural beliefs were measured by six items, such as, 'To accept to play this proposed role would allow me to prioritise health-promotion practices in my duties' (seven-point scale: 7 = strongly agree; 1 = strongly disagree). Facilitating factors were measured with eight items starting with, 'I would accept to play the proposed role if...,' followed by, for example, '...I had the school principal's support' (seven-point scale: 7 = strongly agree; 1 = strongly disagree). Potential barriers were measured with three items, such as, 'I would accept to play the proposed role, despite...,' for instance, '...the nursing shortage' (seven-point scale: 7 = strongly agree; 1 = strongly disagree). Normative beliefs were measured with six items as follows: 'If I accepted to play the proposed role, the following persons would approve/disapprove...' (seven-point scale: 7 = strongly approve; 1 = strongly disapprove). Finally, self-identity was measured by means of four items, such as 'I am a person who is able to negotiate with a different group of persons'.

#### Phase 5: Psychometric qualities

Subsequently, a test-retest using a five-point Likert scale was performed to assess the reliability of the questionnaire with another sample of the studied population. A total of 32 ESNs completed the same version of the questionnaire twice, at two-week intervals. Table 1 presents the internal consistency assessed by means of Cronbach's alpha coefficient [42]; the values varied between .67 and .93. For theoretical variables, this is considered satisfactory for an exploratory study. The temporal stability assessed by means of the intraclass correlation coefficient yielded values varying between .63 and .91, which represent moderate to very good coefficients of agreement [43]. Nonetheless, some behavioural belief items were discarded, and a seven-point Likert-type scale was adapted for the main study in order to increase the variability in responses. The instrument consisted of 40 items, including demographic variables.

**Table 1:** Internal consistency

Variables	Number of items	Alpha coefficients (Cronbach)
		
		(n = 251)
Intention (I)	4	.91^a^

Attitude (Aact)	4	.93^a^

Behavioural beliefs (b)	6	.91

Subjective norm (SN)	3	.88^a^

Normative beliefs (nb)	8	.81

Perceived behavioural control (PBC)	3	.73^a^

Facilitating factors (FF)	8	.84

Barriers (BARR)	3	.84

Personal normative beliefs (PNB)	3	.73

Self-identity (SI)^b^	8	.67

^a^Adjustment for semantic differentiation scales; ^b^n = 250.

### Statistical analyses

Firstly, descriptive analyses of the sample were performed to better describe the variables. A correlational analysis with Pearson coefficients was also carried out between studied variables. A multiple hierarchical linear regression analysis was performed to identify the determinants of intention. This was done as follows: first, past behaviour was entered; second, the proximal variables of the TPB were entered; third, Triandis's variables of moral norm and self-identity were entered; fourth, perceived barriers, facilitating factors, and normative beliefs were entered; and last, sociodemographic variables were added. After each step, variables not reaching *p *< .05 were eliminated from subsequent steps. Finally, a discriminant analysis of the beliefs was performed in order to identify the main targets of action. All analyses were performed using SAS software, Version 9.1 (SAS Institute, Cary, NC, USA) [44].

## Results

Descriptive statistics of the sample are presented in Table 2. The mean age as well as the gender distribution were similar to data on nurses in Québec, namely, 46.1 versus 43.6 years old and 97% versus 90% women, respectively [45]. Three respondents were under 30 years old. A higher proportion of ESNs in our sample held a university diploma (88%) compared to the provincial population of nurses (43%) [45]. The majority held a full-time position (67%). Nonetheless, there was great variability in the time dedicated to elementary school health tasks, since 35% of the participants reported that their working time included tasks such as ad hoc immunization blitzes in schools not under their jurisdiction, youth clinics in hospitals, and perinatal care in the community. The number of schools under an elementary school nurse's responsibility ranged from 1 to 12, and the number of students per ESN was twice the recommended ratio in the United States [46]. Some researchers have suggested that a low percentage of school nurses under 30 years of age may reflect lack of a career pathway and understanding of school nursing [47].

**Table 2:** Sociodemographic characteristics of the participants

Sample characteristics (n = 251)	Frequency
**Gender**	
Male	8 (3%)
Female	243 (97%)
**Mean age**^1 ^(SD)	46.1 (±8.7%)
**Education**	
College diploma	31 (12.4%)
University certificate	41 (16.3%)
Nursing degree	163 (64.9%)
Nursing superior studies (one-year certificate, Msc.)	16 (6.4%)
**Mean years of ESN practice**	
<1 year	30 (11.9%)
1 to 10 years	142 (56.6%)
11 to 20 years	46 (18.3%)
>21 years	33 (13.2%)
**Employment status**^2^	
Full-time	167 (66.8%)
Part-time	66 (26.4%)
On-call	17 (6.8%)
**Mean students per ESN**^3 ^(SD)	1,341 (20-3,400)
**Mean schools per ESN**^4 ^(SD)	5 (1-12)

^1^n = 249; ^2^n = 250; ^3^n = 231; ^4^n = 241. SD = standard deviation; ESN = elementary school nurse.

### Prediction of intention

An examination of the correlation matrix indicated that all psychosocial variables were correlated to intention. Attitude, perceived control, and subjective norm equally presented the greatest association with intention (*r *= .78; *p *< .0001). Tests for multicollinearity were performed and none was detected. Variance inflation factors (VIF) were well below 10, and the condition index was under 30. According to Kline [48], multicollinearity is present when the correlation between two independent variables is greater than .85; none of the coefficients of correlation between the independent variables reached that level. Furthermore, residuals must be normally distributed [49]. Indeed, residual distribution followed a normal curve. An analysis of proportion showed that 73.1% of ESNs indicated moderate to strong intentions to play the role, which means they scored between 5 and 7 on the seven-point Likert-type scale used. With regard to the prediction model, moral norm added to the TPB constructs to predict school nurses' intention. Table 3 shows the steps applied for the multiple linear regression analysis. The strongest determinant of intention was perceived behavioural control (β = 0.36), followed by moral norm (β = 0.27), attitude (β = 0.24), and subjective norm (β = 0.21). The final model explained 83% of the variance in the intention of ESNs to adopt the proposed redefined health-promotion role in the context of the HPS approach in Québec.

**Table 3:** Final predictive model

	Standardised betas								
**Variables entered**	**Model 1**	**Model 2**	**Model 3**	**Model 4**	**Model 5**	**Model 6**	**Model 7**	**Model 8**	**Final model**

Past behaviour	.18**	.00	_	_	_	_	_	_	_
Attitude (Aact)		.36***	.24***	.23***	.24***	.23***	.24***	.24***	.24***
Subjective norm (SN)		.28***	.21***	.21***	.21***	.21***	.22***	.21***	.21***
Perceived behavioural control (PBC)		.38***	.36***	.35***	.35***	.36***	.36***	.35***	.36***
Moral norm (MN)			.27***	.28***	.27***	.27***	.28***	.27***	.27***
Self-identity (SI)				-.05	_	_	_	_	**_**
Perceived barriers (BARR)					.02	_	_	_	**_**
Facilitating factors (FF)						.03	_	_	**_**
Normative beliefs (NB)							-.03	_	**_**
Education								.03	**_**
*R*^2^	.03	.79	.83	.83	.82	.83	.82	.83	**.83**

*p < .05; **p < .001; ***p < .0001.

### Analysis of beliefs

The variables retained for analyses were the salient underlying beliefs from proximal constructs for which a significant relation with intention was identified (*e.g.*, barriers and facilitating factors for perceived behavioural control, behavioural beliefs for attitude, and normative beliefs for subjective norm). In order to identify the beliefs that will serve to guide proper actions, a discriminant analysis contrasting high and low intenders was performed. The results indicated that the item 'This role would allow me to be valued in the performance of my duties' explained the greatest portion of the variance (*R*^2 ^= .17; *p *< .0001). Additional items that also contributed to this prediction were 'If I agreed to play the proposed role, school nurses would approve' (*R*^2 ^= .07; *p *< .0001); 'I would be able to play this role despite the nursing shortage' (*R*^2 ^= .04; *p *< .003); 'If I agreed to play the proposed role, the parents of the students would approve' (*R*^2 ^= .03; *p *< .008); 'To accept to play the role proposed would require me to have leadership' (*R*^2 ^= .02; *p *< .04); and 'This role would allow me to gain a better understanding of school needs' (*R*^2 ^= .02; *p *< .04). These six items represented the underlying beliefs distinguishing those who had a high intention from those who had a low intention (Wilks's lambda = .83; *F *[1] 239 = 50.29; *p *< .0001).

## Discussion

Results suggest that this extended version of the TPB was relevant to predicting elementary school nurses' intention. Indeed, the proportion of the explained variance was noteworthy. In the present study, the strongest determinants of intention were, respectively, perception of behavioural control, moral norm, attitude, and subjective norm.

With respect to the perception of behavioural control, two aspects must be considered: (1) the freedom ESNs have in the decision to agree or not to adopt the role and (2) perceived self-efficacy or perceived competence, both personal and professional, to play the proposed health-promotion role. The first aspect follows Ajzen's definition of perceived control, whereas the second aspect reflects Bandura's self-efficacy construct [50]. With respect to freedom of choice, the decision to adopt such a role is an administrative decision, regardless of the level of intention. For example, protective mandates, such as immunizations, are ruled as mandatory for the studied population. However, when considering the self-efficacy aspect, high and low intenders differed. Respondents who perceived they could overcome barriers, such as the nursing shortage, had a stronger intention to agree to play the health-promotion role, and our findings show that perceived control was highly correlated to moral norm and attitude. Thus, considering that health promotion is a major reason motivating nurses to work in school health [2,3,51] and that health-promotion roles correspond to the values and principles of public health nurses [52], it is plausible that values towards health promotion and perceived advantages led high intenders to believe that they could overcome the nursing shortage. Moreover, Pearcey [53] found that role shifting needs to fit with values and principles espoused by nurses to avoid role confusion. On the other hand, low intenders may be reluctant to adopt such a role because, historically, the nursing shortage has often resulted in lower school nurse staffing with extra workload rather than a reorganisation of mandates, leaving school nurses with poor feelings of self-efficacy to accomplish health-promotion mandates [3,14].

Attitude was another variable explaining the intention of school nurses towards this role. Low and high intenders differed in three perceived advantages or consequences to adopting the proposed role. In order of importance, nurses perceived that this health-promotion role would allow them to feel valued in the performance of their duties. The feeling of being undervalued, especially by their peers, is a recurrent theme from school nurses [2,6,10,11,54,55]. A systematic review on health promotion and the role of school nurses showed that perceived worth is a constraint to the success of school nursing [10]. Smith and Firminn [11] reported that nurses in care settings, as a group, are held in greater respect and value, and conversely, school nurses isolated from nurse colleagues in a milieu are not well recognised by the nursing core. Explanations for this seem to be twofold: First, health outcomes for children are not always tangible in the short-term and the lack of evaluation of their health-promotion practices makes it hard to demonstrate the effectiveness of their work. Second, the difficulty for school nurses have in marketing their role results in a limited understanding of their work by the school system and the nursing community [15,56].

Our findings also raised the leadership issue. Leadership is recognised as a skill that impacts the capacity of nurses to play an expanded health-promotion role at school, since nurses work in professional isolation with minimal resources in an educational sector [6,57-59]. In their study, Morberg *et al. *[6] and Resha [58] found that the absence of clear formal goals for school healthcare and the lack of organisational resources were perceived as having an impact on school nurses' leadership. Leadership encompasses skills such as the delegation of tasks and the ability to market one's role [56,57,60-63]. Difficulties delegating tasks in a health-promotion role in expansion are also associated with frustration among school nurses and inefficiency [17].

A third underlying belief of the school nurses' attitude is the perceived advantage of gaining a better understanding of school needs. Resha [58] reported that a limited understanding of schools as an organisation was a barrier to school nurses' ability to exercise leadership in a health-promotion role. In New Zealand, Kool and colleagues [4] found that school nurses who chose to adopt a role redefined in health-promotion terms instead of their actual traditional role believed this option helped them to gain more knowledge and a better understanding of school needs. Thus, it seems that better knowledge of school needs, leadership, and feelings of worth are linked.

The subjective norm was a significant factor in explaining school nurses' intention to adopt the proposed role. This means that the participants are likely to be influenced by the perceived expectations of significant others. Our findings indicate that school nurses consider parents to be significant in their rapport with the children. Thus, the adoption of the proposed role could enhance the relationship between nurses and the parents of students. Parental approval is important, considering the age groups of the children under ESN care and the need for local support to improve the nurse to student ratio, for example [5,64]. The school nurse may perceive that the proposed role would allow a wider scope of action, thus be more visible to parents. The last significant advantage perceived by the respondents is that school nurses as a whole would approve of playing this role. Thus, it reaffirms the motivation of ESNs for the role.

None of the sociodemographic variables predicted elementary school nurses' intention. This finding contrasts with the literature, where employment status for health-promotion role, ratio of ESNs to students, number of schools per ESN, and ESN educational levels are reported as critical factors in the individual decision to adopt a health-promotion role [2,5,17,65]. Training needs were not expressed as a facilitating factor to playing the proposed role by the respondents, although there is a consensus in the literature on the development of competencies with respect to an expanded role of school nurses [17,18,55,66]. In their study, Beaudet *et al. *[13] noted that nurses tended to mention that they needed training, but when questioned about the nature, they had difficulty identifying required training and competencies. The authors attributed this to the fact that public nurses tend to limit their educational needs to the individual and family. Nonetheless, our findings are consistent with TPB assumptions, which views the influence of such variables as mediated by the TPB variables defining intention.

Finally, from a theoretical point of view, it would be interesting to have more studies relying on theoretical foundations for the identification of intention as well as behavioural determinants of the adoption by nurses of health-promotion roles. Indeed, the literature is mainly anecdotal, and the rare quantitative studies are based on small sample sizes and not always explicit about their psychometric qualities. Qualitative studies are more frequent but few of them discuss their quality criteria.

### Implications for the adoption of a health-promotion role in the context of the health-promoting school by school nurses

For managers and administrators, it is valuable information to know that approval by parents and school nurses, increased feeling of valorisation, and increased knowledge of school needs would motivate school nurses to play such a role. These beliefs are factual information of worth to use to market the role. MacDonald and Schoenfeld [66] found that public health nurses' involvement in research and use of their input in planning, delivering, and evaluating health-promotion programs promoted a sense of achievement, increased feelings of being valued, and greater respect from other professionals. Others also demonstrated that positive outcomes for students contributed to feelings of worth [10,67]. The role proposed implies that nurses become involved in research. Thus, their involvement in research linking their health-promoting actions to child health outcomes, child well-being, and academic outcomes would likely contribute to feelings of worth and produce evidence-based knowledge that can promote their role as decision makers [56].

Findings also indicate that a nursing shortage and leadership require that action be taken. High intenders feel that they could overcome barriers such as the nursing shortage. However, this finding needs to be interpreted carefully by health administrators in order to avoid burnout among these human resources. In fact, if supervisors want to increase the proportion of high intenders, they need to ensure adequate nurse staffing in schools, as a nursing shortage is recognised as a serious threat to the deployment of health-promotion practices by nurses [52]. Indeed, a shortage of nurses means understaffing and decreased presence in the milieu. The success of the HPS approach depends on the stability of networks over time [68], and networking requires a minimum of shared time among the different actors [13]. A presence at school is also associated with leadership. Leadership has been shown to be a key component to improving nurse staffing and retention, as well as health-promotion practices [57]. In the international context of financial constraints, where additional nursing staffing has proven difficult to obtain, investing in the development of delegation skills could help school nurses increase their presence where needed to advance a role in health promotion. It would also contribute to heightened feelings of worth [63]. Leadership is a major skill that should be addressed at the university level, preferably in the context of a school health specialisation, so that school nurses are socialised to be leaders and expected to act as leaders before they enter the school system. Leadership training could also be provided to school nurses in the form of workshops in collaboration with local universities. Leadership development covers topics such as management of resources, marketing, team motivation, negotiation, effective communication, organisational change, contribution to the development of policies, mentoring, and delegation [57].

This study also shows that ESNs do not form a homogeneous population and that individual considerations should be taken into account for the implementation of interventions planned for the traditional and the innovative type of ESN. The latter seems to rely more on individual resources, while the former tends to rely more on organisational resources [4].

### Study limitations

This study presents some limitations. Even though the sample accounts for a large portion of the ESN population, only local health organisations with five ESNs or more were invited to participate in order to ensure the required number of participants for a multilevel analysis [69]. Thus, smaller regional sites were not included in the present study. Furthermore, the sample was composed of volunteers. Therefore, responses to this study are subject to self-selection biases. Also, it may have been difficult for participants to determine their intention regarding the hypothetic role, since none of them has played this precise role in the past. There is also a potential influence of the social desirability bias. Thus, some caution should be exercised before generalising the results.

## Summary

To the best of our knowledge, this study was the first to apply an extended version of the TPB to investigate the determinants of elementary school nurses' intention to adopt a redefined health-promotion role proposed to them. As such, this study is among the rarest to produce knowledge that is theoretically based on this subject. The international interest for an expanded role of the school nurse in health promotion, combined with the anticipated proximal massive retirement of ESNs, can be seen as a window of opportunity to redefine this role in an optimal way. Since the school nurse role proposed by us corresponds to similar expectations with respect to health promotion in many countries, we believe our findings bring evidence-based knowledge that can inform other school health programs.

Our results show that the lack of leadership skills and the nursing shortage are targets that administrators can work on to raise the proportion of high intenders among school nurses and to advance them towards an expanded role. The development of leadership among school nurses could contribute to alleviating nursing shortage effects and increase feelings of worth, as it encompasses delegation and marketing skills, known to be key components of effectiveness, efficiency, and school nurse satisfaction [63]. Finally, our findings indicate the need to study organisational factors in order to explore more extensively potential contextual determinants influencing the adoption of this role, such as resources and policies. Experimental research with regard to leadership training effects is recommended.

## Competing interests

The authors declare that they have no competing interests.

## Authors' contributions

GC and GG planned the study. GC conducted and supervised the entire study. GC drafted the manuscript and GG and M-PG reviewed it. All authors read and approved the final manuscript.

## Supplementary Material

Additional file 1 **Questionnaire**. A copy of the questionnaire used in the study. Click here for file
