# Supplementary material for: Determinants of the intention of elementary school nurses to adopt a redefined role in health promotion at school
Source: Implement Sci. 2010 Nov 26;5:93. doi: 10.1186/1748-5908-5-93 (PMC3003233; doi:10.1186/1748-5908-5-93)
Supplement: Additional file 1 — Questionnaire. A copy of the questionnaire used in the study. [file 1748-5908-5-93-S1.DOC]

##
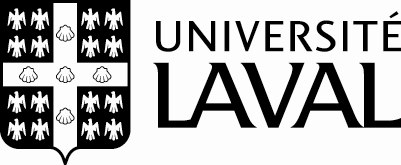


# **DÉTERMINANTS PSYCHOSOCIAUX ET ORGANISATIONNELS DE L’ADOPTION D’UN RÔLE INFIRMIER REDÉFINI EN MILIEU SCOLAIRE PRIMAIRE DANS LE CONTEXTE D’« ÉCOLES ET MILIEUX EN SANTÉ »**

## ***Questionnaire***

Date : **________________**

jj / mm / aaaa

|  |  |  |
| --- | --- | --- |

# Certaines questions peuvent vous sembler répétitives. Toutefois, il est très important de **répondre à toutes les questions**.

# Pour répondre aux questions, vous devez inscrire votre réponse à l’endroit reflétant le mieux **votre opinion** **sur l’ensemble du rôle proposé**

# **Exemple**

# Il y a beaucoup de neige au Québec en hiver.

|  |  |  |  |  |  |  |  |  |  |  | **✓** |  |  |
| --- | --- | --- | --- | --- | --- | --- | --- | --- | --- | --- | --- | --- | --- |
|  | Fortement en désaccord |  | Assez en désaccord |  | Légèrement en désaccord |  | Ni en désaccord  ni en accord |  | Légèrement en accord |  | Assez en accord |  | Fortement en accord |

# Prenez le temps de lire attentivement chaque question ou énoncé et **donnez la réponse reflétant le mieux ce que vous pensez actuellement.**

# Il n’y a ni bonne ni mauvaise réponse.

# Vos réponses demeureront confidentielles.

# **DÉTERMINANTS PSYCHOSOCIAUX ET ORGANISATIONNELS DE L’ADOPTION D’UN RÔLE INFIRMIER REDÉFINI EN MILIEU SCOLAIRE PRIMAIRE DANS LE CONTEXTE D’« ÉCOLES ET MILIEUX EN SANTÉ »**

L'approche *Écoles et milieux en santé* (ÉMES) repose sur l'idée qu'il est judicieux d'agir globalement sur un ensemble de facteurs susceptibles d’exercer un impact positif sur la santé des jeunes. La mise en place de cette approche invite à revoir les pratiques et les formes de partenariats sur les plans régional et local. L'approche ÉMES invite également à créer des conditions organisationnelles favorables à son déploiement.

Dans le contexte d’ÉMES, le rôle infirmier proposé consiste à occuper des fonctions visant :

| a) Le changement planifié des habitudes de vie : Ex. Alimentation, activité physique, tabagisme, etc. |
| --- |
| *b) Le changement planifié des conditions de vie :*  Service de santé (préventifs et curatifs) pour les élèves et le personnel de l’école *  Implication dans l’environnement social et physique de l’école :  Favoriser une implication précoce des jeunes et de leur famille dans les interventions  Participer aux réunions de l’équipe-école et du conseil d’établissement  Planifier, coordonner et participer à l’évaluation d’*Écoles et milieux en santé* dans mon milieu  Collaborer avec les acteurs du milieu : scolaire, municipal et communautaire  Participer au développement de politiques favorables à la santé des jeunes en milieu scolaire primaire  Participer au développement des politiques infirmières en milieu scolaire  Participer à l’élaboration des questions de recherche concernant la santé des jeunes et la pratique infirmière en santé scolaire  Assurer une représentation à la Table de concertation jeunesse  Faire connaître son rôle aux jeunes, à leur famille, au milieu scolaire et à la population  Utiliser des données de recherche visant la résolution de problèmes de santé et de développement des jeunes pour l’intervention, la formation et la consultation auprès des jeunes, de leur famille et de l’équipe jeunesse  Maintenir un registre des pratiques de promotion de la santé qui ont un impact sur la santé des jeunes |
| * Planification et coordination des cliniques de vaccination et suivi des carnets de santé, mais délégation de l’acte vaccinal |

1. Avez-vous déjà occupé un poste qui demandait de réaliser ces fonctions en milieu scolaire ?

□ Toutes ces fonctions

□ La plupart de ces fonctions

□ Quelques-unes de ces fonctions

□ Aucune de ces fonctions

Précisez

**Pour les questions qui suivent, placez-vous dans une situation où on vous**

**proposerait d’occuper l’ensemble de ce rôle dans le contexte d’ÉMES**

1. Dans le contexte d’ÉMES, si on m’offrait de jouer le rôle proposé, mon intention serait de l’accepter

|  |  |  |  |  |  |  |  |  |  |  |  |  |  |
| --- | --- | --- | --- | --- | --- | --- | --- | --- | --- | --- | --- | --- | --- |
|  | Très improbable |  | Assez  improbable |  | Légèrement  improbable |  | Ni improbable  ni probable |  | Légèrement  probable |  | Assez probable |  | Très  probable |

1. La plupart des personnes qui sont importantes pour moi me recommanderaient d’accepter de jouer le

rôle proposé dans le contexte d’ÉMES

|  |  |  |  |  |  |  |  |  |  |  |  |  |  |
| --- | --- | --- | --- | --- | --- | --- | --- | --- | --- | --- | --- | --- | --- |
|  | Fortement en désaccord |  | Assez en désaccord |  | Légèrement en désaccord |  | Ni en désaccord  ni en accord |  | Légèrement en accord |  | Assez en accord |  | Fortement en accord |

1. Dans le contexte d’ÉMES, accepter de jouer le rôle proposé fait partie de mes valeurs

|  |  |  |  |  |  |  |  |  |  |  |  |  |  |
| --- | --- | --- | --- | --- | --- | --- | --- | --- | --- | --- | --- | --- | --- |
|  | Fortement en désaccord |  | Assez en désaccord |  | Légèrement en désaccord |  | Ni en désaccord  ni en accord |  | Légèrement en accord |  | Assez en accord |  | Fortement en accord |

1. **POUR MOI, ACCEPTER DE JOUER LE RÔLE PROPOSÉ DANS LE CONTEXTE D’ÉMES SERAIT :**

*[Cochez la case appropriée pour* ***chacun des quatre qualificatifs suivants (5.1 à 5.4)****]*

| 5.1 |  |  |  |  |  |  |  |  |  |  |  |  |  |
| --- | --- | --- | --- | --- | --- | --- | --- | --- | --- | --- | --- | --- | --- |
|  | Très désagréable |  | Assez  désagréable |  | Légèrement  désagréable |  | Ni désagréable  ni agréable |  | Légèrement  agréable |  | Assez agréable |  | Très  agréable |

| 5.2 |  |  |  |  |  |  |  |  |  |  |  |  |  |
| --- | --- | --- | --- | --- | --- | --- | --- | --- | --- | --- | --- | --- | --- |
|  | Très dévalorisant |  | Assez  dévalorisant |  | Légèrement  dévalorisant |  | Ni dévalorisant  ni valorisant |  | Légèrement  valorisant |  | Assez valorisant |  | Très  valorisant |

| 5.3 |  |  |  |  |  |  |  |  |  |  |  |  |  |
| --- | --- | --- | --- | --- | --- | --- | --- | --- | --- | --- | --- | --- | --- |
|  | Très  inutile |  | Assez  inutile |  | Légèrement  inutile |  | Ni inutile  ni utile |  | Légèrement  utile |  | Assez  utile |  | Très  utile |

| 5.4 |  |  |  |  |  |  |  |  |  |  |  |  |  |
| --- | --- | --- | --- | --- | --- | --- | --- | --- | --- | --- | --- | --- | --- |
|  | Très déplaisant |  | Assez  déplaisant |  | Légèrement  déplaisant |  | Ni déplaisant  ni plaisant |  | Légèrement  plaisant |  | Assez plaisant |  | Très  plaisant |

1. **POUR MOI, ACCEPTER DE JOUER LE RÔLE PROPOSÉ DANS LE CONTEXTE D’ÉMES …**

*[Cochez la case appropriée pour* ***chacun des six énoncés suivants (6.1 à 6.6)****]*

|  | **Très en désaccord** | **Assez en désaccord** | **Légèrement en désaccord** | **Ni l’un**  **ni l’autre** | **Légèrement en accord** | **Assez en accord** | **Très en accord** |
| --- | --- | --- | --- | --- | --- | --- | --- |
| - 1. M’obligerait à avoir du   leadership | □ | □ | □ | □ | □ | □ | □ |
| - 1. Me permettrait de jouer un rôle pivot dans le milieu | □ | □ | □ | □ | □ | □ | □ |
| - 1. Clarifierait mon rôle | □ | □ | □ | □ | □ | □ | □ |
| - 1. Me permettrait d’être valorisé(e) dans l’exercice de mes fonctions | □ | □ | □ | □ | □ | □ | □ |
| - 1. Me permettrait d’avoir une meilleure connaissance des besoins du milieu scolaire | □ | □ | □ | □ | □ | □ | □ |
| - 1. Me permettrait de prioriser les pratiques de promotion de la santé dans mon travail | □ | □ | □ | □ | □ | □ | □ |

**DANS LE CONTEXTE D’ÉMES, JE SERAIS CAPABLE D’ACCEPTER DE JOUER LE RÔLE PROPOSÉ, MALGRÉ :**

1. La pénurie d’infirmières

|  |  |  |  |  |  |  |  |  |  |  |  |  |  |
| --- | --- | --- | --- | --- | --- | --- | --- | --- | --- | --- | --- | --- | --- |
|  | Fortement en désaccord |  | Assez en désaccord |  | Légèrement en désaccord |  | Ni en désaccord  ni en accord |  | Légèrement en accord |  | Assez en accord |  | Fortement en accord |

1. Le manque de temps pour assumer correctement toutes les tâches proposées

|  |  |  |  |  |  |  |  |  |  |  |  |  |  |
| --- | --- | --- | --- | --- | --- | --- | --- | --- | --- | --- | --- | --- | --- |
|  | Fortement en désaccord |  | Assez en désaccord |  | Légèrement en désaccord |  | Ni en désaccord  ni en accord |  | Légèrement en accord |  | Assez en accord |  | Fortement en accord |

1. Le manque de ressources matérielles et didactiques (ordinateur, Internet, cellulaire…)

|  |  |  |  |  |  |  |  |  |  |  |  |  |  |
| --- | --- | --- | --- | --- | --- | --- | --- | --- | --- | --- | --- | --- | --- |
|  | Fortement en désaccord |  | Assez en désaccord |  | Légèrement en désaccord |  | Ni en désaccord  ni en accord |  | Légèrement en accord |  | Assez en accord |  | Fortement en accord |

1. **Je suis une personne qui :**
   1. A du leadership

|  |  |  |  |  |  |  |  |  |  |  |  |  |  |
| --- | --- | --- | --- | --- | --- | --- | --- | --- | --- | --- | --- | --- | --- |
|  | Fortement en désaccord |  | Assez en désaccord |  | Légèrement en désaccord |  | Ni en désaccord  ni en accord |  | Légèrement en accord |  | Assez en accord |  | Fortement en accord |

- 1. Est innovatrice

|  |  |  |  |  |  |  |  |  |  |  |  |  |  |
| --- | --- | --- | --- | --- | --- | --- | --- | --- | --- | --- | --- | --- | --- |
|  | Fortement en désaccord |  | Assez en désaccord |  | Légèrement en désaccord |  | Ni en désaccord  ni en accord |  | Légèrement en accord |  | Assez en accord |  | Fortement en accord |

- 1. Est capable de négocier avec différents groupes de personnes

|  |  |  |  |  |  |  |  |  |  |  |  |  |  |
| --- | --- | --- | --- | --- | --- | --- | --- | --- | --- | --- | --- | --- | --- |
|  | Fortement en désaccord |  | Assez en désaccord |  | Légèrement en désaccord |  | Ni en désaccord  ni en accord |  | Légèrement en accord |  | Assez en accord |  | Fortement en accord |

- 1. Fait une distinction entre la prévention et la promotion de la santé

|  |  |  |  |  |  |  |  |  |  |  |  |  |  |
| --- | --- | --- | --- | --- | --- | --- | --- | --- | --- | --- | --- | --- | --- |
|  | Fortement en désaccord |  | Assez en désaccord |  | Légèrement en désaccord |  | Ni en désaccord  ni en accord |  | Légèrement en accord |  | Assez en accord |  | Fortement en accord |

1. **Un(e) infirmier(e) en milieu scolaire primaire qui accepterait de jouer le rôle proposé devrait être une personne qui :**
2. A du leadership

|  |  |  |  |  |  |  |  |  |  |  |  |  |  |
| --- | --- | --- | --- | --- | --- | --- | --- | --- | --- | --- | --- | --- | --- |
|  | Fortement en désaccord |  | Assez en désaccord |  | Légèrement en désaccord |  | Ni en désaccord  ni en accord |  | Légèrement en accord |  | Assez en accord |  | Fortement en accord |

1. Est innovatrice

|  |  |  |  |  |  |  |  |  |  |  |  |  |  |
| --- | --- | --- | --- | --- | --- | --- | --- | --- | --- | --- | --- | --- | --- |
|  | Fortement en désaccord |  | Assez en désaccord |  | Légèrement en désaccord |  | Ni en désaccord  ni en accord |  | Légèrement en accord |  | Assez en accord |  | Fortement en accord |

1. Est capable de négocier avec différents groupes de personnes

|  |  |  |  |  |  |  |  |  |  |  |  |  |  |
| --- | --- | --- | --- | --- | --- | --- | --- | --- | --- | --- | --- | --- | --- |
|  | Fortement en désaccord |  | Assez en désaccord |  | Légèrement en désaccord |  | Ni en désaccord  ni en accord |  | Légèrement en accord |  | Assez en accord |  | Fortement en accord |

1. Fait une distinction entre la prévention et la promotion de la santé

|  |  |  |  |  |  |  |  |  |  |  |  |  |  |
| --- | --- | --- | --- | --- | --- | --- | --- | --- | --- | --- | --- | --- | --- |
|  | Fortement en désaccord |  | Assez en désaccord |  | Légèrement en désaccord |  | Ni en désaccord  ni en accord |  | Légèrement en accord |  | Assez en accord |  | Fortement en accord |

**Pour les questions qui suivent, placez-vous dans une situation où on vous**

**proposerait d’occuper la totalité de ce rôle dans le contexte d’ÉMES**

1. Pour moi, accepter de jouer le rôle proposé dans le contexte d’ÉMES serait

|  |  |  |  |  |  |  |  |  |  |  |  |  |  |
| --- | --- | --- | --- | --- | --- | --- | --- | --- | --- | --- | --- | --- | --- |
|  | Très  difficile |  | Assez difficile |  | Légèrement difficile |  | Ni difficile  ni facile |  | Légèrement facile |  | Assez  facile |  | Très  facile |

1. Dans le contexte d’ÉMES, j’évalue que mes chances d’accepter de jouer le rôle proposé seraient…

|  |  |  |  |  |  |  |  |  |  |  |  |  |  |
| --- | --- | --- | --- | --- | --- | --- | --- | --- | --- | --- | --- | --- | --- |
|  | Très  faibles |  | Assez  faibles |  | Légèrement  faibles |  | Ni faibles  ni fortes |  | Légèrement  fortes |  | Assez  fortes |  | Très  fortes |

1. Dans le contexte d’ÉMES, je me sentirais COUPABLE de NE PAS accepter de jouer le rôle proposé

|  |  |  |  |  |  |  |  |  |  |  |  |  |  |
| --- | --- | --- | --- | --- | --- | --- | --- | --- | --- | --- | --- | --- | --- |
|  | Fortement en désaccord |  | Assez en désaccord |  | Légèrement en désaccord |  | Ni en désaccord  ni en accord |  | Légèrement en accord |  | Assez en accord |  | Fortement en accord |

1. Si j’acceptais de jouer le rôle proposé dans le contexte d’ÉMES, la plupart des personnes qui sont

importantes pour moi

|  |  |  |  |  |  |  |  |  |  |  |  |  |  |
| --- | --- | --- | --- | --- | --- | --- | --- | --- | --- | --- | --- | --- | --- |
|  | Désapprouverait fortement |  | Désapprouverait assez |  | Désapprouverait légèrement |  | Ni désapprouverait  ni approuverait |  | Approuverait légèrement |  | Approuverait assez |  | Approuverait fortement |

1. Jusqu’à quel point croyez-vous exercer un contrôle sur le fait d’accepter de jouer le rôle proposé

dans le contexte d’ÉMES ?

|  |  |  |  |  |  |  |  |  |  |  |  |  |  |
| --- | --- | --- | --- | --- | --- | --- | --- | --- | --- | --- | --- | --- | --- |
|  | Très  incontrôlable |  | Assez incontrôlable |  | Légèrement incontrôlable |  | Ni incontrôlable  ni contrôlable |  | Légèrement  contrôlable |  | Assez  contrôlable |  | Très  contrôlable |

**DANS LE CONTEXTE D’ÉMES, J’ACCEPTERAIS DE JOUER LE RÔLE PROPOSÉ SI :**

1. Ma formation de base était mieux adaptée

|  |  |  |  |  |  |  |  |  |  |  |  |  |  |
| --- | --- | --- | --- | --- | --- | --- | --- | --- | --- | --- | --- | --- | --- |
|  | Fortement en désaccord |  | Assez en désaccord |  | Légèrement en désaccord |  | Ni en désaccord  ni en accord |  | Légèrement en accord |  | Assez en accord |  | Fortement en accord |

1. Si on m’offrait une formation pertinente en cours d’emploi (ex. gestion de projets, programmation…)

|  |  |  |  |  |  |  |  |  |  |  |  |  |  |
| --- | --- | --- | --- | --- | --- | --- | --- | --- | --- | --- | --- | --- | --- |
|  | Fortement en désaccord |  | Assez en désaccord |  | Légèrement en désaccord |  | Ni en désaccord  ni en accord |  | Légèrement en accord |  | Assez en accord |  | Fortement en accord |

1. Le ratio infirmière/école était amélioré

|  |  |  |  |  |  |  |  |  |  |  |  |  |  |
| --- | --- | --- | --- | --- | --- | --- | --- | --- | --- | --- | --- | --- | --- |
|  | Fortement en désaccord |  | Assez en désaccord |  | Légèrement en désaccord |  | Ni en désaccord  ni en accord |  | Légèrement en accord |  | Assez en accord |  | Fortement en accord |

1. J’avais le soutien de la direction d’école

|  |  |  |  |  |  |  |  |  |  |  |  |  |  |
| --- | --- | --- | --- | --- | --- | --- | --- | --- | --- | --- | --- | --- | --- |
|  | Fortement en désaccord |  | Assez en désaccord |  | Légèrement en désaccord |  | Ni en désaccord  ni en accord |  | Légèrement en accord |  | Assez en accord |  | Fortement en accord |

1. J’étais assuré(e) de la collaboration des enseignants

|  |  |  |  |  |  |  |  |  |  |  |  |  |  |
| --- | --- | --- | --- | --- | --- | --- | --- | --- | --- | --- | --- | --- | --- |
|  | Fortement en désaccord |  | Assez en désaccord |  | Légèrement en désaccord |  | Ni en désaccord  ni en accord |  | Légèrement en accord |  | Assez en accord |  | Fortement en accord |

1. J’étais présent(e) 5 jours par semaine dans la même école

|  |  |  |  |  |  |  |  |  |  |  |  |  |  |
| --- | --- | --- | --- | --- | --- | --- | --- | --- | --- | --- | --- | --- | --- |
|  | Fortement en désaccord |  | Assez en désaccord |  | Légèrement en désaccord |  | Ni en désaccord  ni en accord |  | Légèrement en accord |  | Assez en accord |  | Fortement en accord |

1. Je pouvais déléguer certaines tâches (ex. vaccination, statistiques, tâches cléricales, premiers soins...)

|  |  |  |  |  |  |  |  |  |  |  |  |  |  |
| --- | --- | --- | --- | --- | --- | --- | --- | --- | --- | --- | --- | --- | --- |
|  | Fortement en désaccord |  | Assez en désaccord |  | Légèrement en désaccord |  | Ni en désaccord  ni en accord |  | Légèrement en accord |  | Assez en accord |  | Fortement en accord |

1. Les décideurs priorisaient un rôle de promotion-prévention pour les infirmières

|  |  |  |  |  |  |  |  |  |  |  |  |  |  |
| --- | --- | --- | --- | --- | --- | --- | --- | --- | --- | --- | --- | --- | --- |
|  | Fortement en désaccord |  | Assez en désaccord |  | Légèrement en désaccord |  | Ni en désaccord  ni en accord |  | Légèrement en accord |  | Assez en accord |  | Fortement en accord |

1. SI J’ACCEPTAIS DE JOUER LE RÔLE PROPOSÉ DANS LE CONTEXTE D’ÉMES, LES PERSONNES SUIVANTES APPROUVERAIENT/DÉSAPPROUVERAIENT :

|  | Désapprouverait fortement | Désapprouverait assez | Désapprouverait légèrement | Ni désapprouverait  ni approuverait | Approuverait légèrement | Approuverait assez | Approuverait fortement |
| --- | --- | --- | --- | --- | --- | --- | --- |
| 25.1 Les directions d’école | □ | □ | □ | □ | □ | □ | □ |
| 25.2 Les enseignants | □ | □ | □ | □ | □ | □ | □ |
| 25.3 Mes gestionnaires | □ | □ | □ | □ | □ | □ | □ |
| 25.4 Les parents | □ | □ | □ | □ | □ | □ | □ |
| 25.5 Les infirmières scolaires | □ | □ | □ | □ | □ | □ | □ |
| 25.6 Les élèves | □ | □ | □ | □ | □ | □ | □ |
| 25.7 L’OIIQ | □ | □ | □ | □ | □ | □ | □ |
| 25.8 Le syndicat | □ | □ | □ | □ | □ | □ | □ |

1. Dans le contexte d’ÉMES, j’accepterais de jouer le rôle proposé si on m’en donnait l’opportunité

|  |  |  |  |  |  |  |  |  |  |  |  |  |  |
| --- | --- | --- | --- | --- | --- | --- | --- | --- | --- | --- | --- | --- | --- |
|  | Très improbable |  | Assez  improbable |  | Légèrement  improbable |  | Ni improbable  ni probable |  | Légèrement  probable |  | Assez probable |  | Très  probable |

1. Je suis confiant(e) d’avoir la capacité de surmonter les obstacles qui pourraient m’empêcher d’accepter de jouer le rôle proposé dans le contexte d’ÉMES

|  |  |  |  |  |  |  |  |  |  |  |  |  |  |
| --- | --- | --- | --- | --- | --- | --- | --- | --- | --- | --- | --- | --- | --- |
|  | Fortement en désaccord |  | Assez en désaccord |  | Légèrement en désaccord |  | Ni en désaccord  ni en accord |  | Légèrement en accord |  | Assez en accord |  | Fortement en accord |

1. Les personnes les plus importantes pour moi pensent que je devrais accepter d’occuper le rôle proposé dans le contexte d’ÉMES

|  |  |  |  |  |  |  |  |  |  |  |  |  |  |
| --- | --- | --- | --- | --- | --- | --- | --- | --- | --- | --- | --- | --- | --- |
|  | Fortement en désaccord |  | Assez en désaccord |  | Légèrement en désaccord |  | Ni en désaccord  ni en accord |  | Légèrement en accord |  | Assez en accord |  | Fortement en accord |

1. Dans le contexte d’ÉMES, il est dans mes principes d’accepter de jouer le rôle proposé

|  |  |  |  |  |  |  |  |  |  |  |  |  |  |
| --- | --- | --- | --- | --- | --- | --- | --- | --- | --- | --- | --- | --- | --- |
|  | Fortement en désaccord |  | Assez en désaccord |  | Légèrement en désaccord |  | Ni en désaccord  ni en accord |  | Légèrement en accord |  | Assez en accord |  | Fortement en accord |

1. Dans le contexte d’ÉMES, je serais capable d’accepter de jouer le rôle proposé

|  |  |  |  |  |  |  |  |  |  |  |  |  |  |
| --- | --- | --- | --- | --- | --- | --- | --- | --- | --- | --- | --- | --- | --- |
|  | Très improbable |  | Assez  improbable |  | Légèrement  improbable |  | Ni improbable  ni probable |  | Légèrement  probable |  | Assez probable |  | Très  probable |

1. Dans le contexte d’ÉMES, si j’avais le choix, j’accepterais de jouer le rôle proposé

|  |  |  |  |  |  |  |  |  |  |  |  |  |  |
| --- | --- | --- | --- | --- | --- | --- | --- | --- | --- | --- | --- | --- | --- |
|  | Très improbable |  | Assez  improbable |  | Légèrement  improbable |  | Ni improbable  ni probable |  | Légèrement  probable |  | Assez probable |  | Très  probable |

1. Quel âge avez-vous ? ______ans
2. Quel est votre sexe ?

🞎 Femme 🞎 Homme

1. Quel est le plus haut niveau de scolarité que vous avez complété ?

🞎 Collégial 🞎 Universitaire (DESS)

🞎 Universitaire (certificat 1^er^ cycle) 🞎 Universitaire (Maîtrise)

🞎 Universitaire (bac)

1. Nombre d’années d’expérience en santé scolaire primaire ?

🞎 Moins d’un an

🞎 Entre 1 et 10 ans

🞎 Entre 11 et 20 ans

🞎 21 ans et +

1. Votre statut d’emploi?

🞎 TPO

🞎 TPR

🞎 TC

37. Vous travaillez dans combien d’écoles primaires ? _____

38. Combien d’élèves au primaire ?________

39. Combien de jours/semaine au primaire ?______

40. Vous travaillez dans combien d’écoles secondaires ? _____ Combien d’élèves au secondaire ?_____

**Assurez-vous d’avoir répondu à toutes les questions.**

**Merci de votre précieuse collaboration !**

## **Si vous avez des commentaires ou suggestions concernant ce questionnaire**

**ou cette recherche, vous pouvez les inscrire**

#### Commentaires

|  |
| --- |
|  |
|  |
|  |
|  |
|  |
|  |
|  |
|  |
|  |
|  |
|  |
|  |
|  |
|  |
|  |
|  |
|  |
